# New Biocomposite Electrospun Fiber/Alginate Hydrogel for Probiotic Bacteria Immobilization

**DOI:** 10.3390/ma14143861

**Published:** 2021-07-10

**Authors:** Adam Grzywaczyk, Agata Zdarta, Katarzyna Jankowska, Andrzej Biadasz, Jakub Zdarta, Teofil Jesionowski, Ewa Kaczorek, Wojciech Smułek

**Affiliations:** 1 Institute of Chemical Technology and Engineering, Faculty of Chemical Technology, Poznan University of Technology, Berdychowo 4, 60-965 Poznan, Poland; adam.grzywaczyk@doctorate.put.poznan.pl (A.G.); agata.zdarta@put.poznan.pl (A.Z.); katarzyna.jankowska@doctorate.put.poznan.pl (K.J.); jakub.zdarta@put.poznan.pl (J.Z.); teofil.jesionowski@put.poznan.pl (T.J.); ewa.kaczorek@put.poznan.pl (E.K.); 2 Institute of Physics, Faculty of Materials Engineering and Technical Physics, Poznan University of Technology, Piotrowo 3, 60-965 Poznan, Poland; andrzej.biadasz@put.poznan.pl

**Keywords:** biocomposite, contact angle, electrospun nanofibers, immobilization, *Lactobacillus*, alginate hydrogel

## Abstract

Biotechnological use of probiotic microorganisms involves providing them with appropriate conditions for growth, but also protection against environmental changes caused by an exchange of the medium, isolation of metabolites, etc. Therefore, the research on effective immobilization of probiotic microorganisms should be focused in this direction. The present study aimed to evaluate the effectiveness of an innovative hybrid immobilization system based on electrospun nanofibers and alginate hydrogel. The analyses carried out included the study of properties of the initial components, the evaluation of the degree and durability of cell immobilization in the final material, and their survival under stress conditions. Effective binding of microorganisms to the hydrogel and nanofibers was confirmed, and the collected results proved that the proposed biocomposite is an efficient method of cell protection. In addition, it was shown that immobilization on electrospun nanofibers leads to the preservation of the highest cell activity and the least cell growth restriction as compared to free or lyophilized cells only. The completed research opens new perspectives for the effective immobilization of microorganisms of significant economic importance.

## 1. Introduction

To maintain optimal microorganism cultivation, growth media parameters such as pH, temperature, oxygen level, and nutrition agents must be ensured, depending on the species of microbe (Thomas, 2015 [1]). This causes the use of many different specific and non-specific growth media, such as general-purpose nutrient agar (Smułek et al., 2020 [2]) and enrichment broth (Rahman et al., 2020 [3]), specific for certain fungi Sabouraud’s agar (Cultrera et al., 2021 [4]), and Middlebrook and Löwenstein-Jensen media used for *Mycobacterium* spp. culture (Schön et al., 2020 [5]; Xie et al., 2021 [6]) (Mikolajczyk et al., 2016 [7]).

No matter what type of growth media is required, it is crucial is to maintain sterility when working with microorganisms, and prevent microbes from hazardous environmental conditions such as temperature fluctuation, UV radiation, pH changes, organic solvents, and salts. One possible way to do this is the immobilization of microorganisms—chemically bonding or adsorbing microorganisms onto the surface of the carrier, and entrapment in a form of capsule or gel (Drauz and Waldmann, 1995 [8]). Requirements for the appropriate carrier, such as non-toxicity, optimal mechanical properties, chemical resistance, and cost-effectiveness make the entrapment in calcium or sodium alginate hydrogels the most-used technique for cell immobilization (Zhu, 2007 [9]). There are two main types of this process: gel entrapment and encapsulation. Gel entrapment differs from encapsulation in the form of beads. During the gel entrapment, cells are immobilized in a gel matrix, whereas the encapsulation process involves the preparation of porous barrier and entrapment of microorganism in liquid core of such bead (Willaert and Baron, 1996 [10]). Probiotics are the group of microorganism which are most commonly stabilized by the process of encapsulation since their survival in harsh conditions is crucial to trigger the beneficial effect of those microorganisms in human body (Tripathi and Giri, 2014 [11]). Moreover, capsuled microbes have been used in the medical application (Rocha et al., 2021 [12]), the food industry (Penhasi, Reuveni and Baluashvili, 2021 [13]), delivery systems (Cook et al., 2012 [14]), or even in larval rearing system (Ghoname et al., 2020 [15]).

When presenting the process of microbial immobilization, the adsorption process on nanofibers should be mentioned. Nanofibers are one-dimensional structures whose cross-sectional diameter is in the nanometric range (from 1 to 100 nm). Due to their structure and morphology, there can be distinguished porous, ribbon, branched, or hollow nanofibers, and each of them is created under specific conditions of their production process (Stanishevsky, Wetuski, and Yockell-Lelièvre, 2016 [16]; Li et al., 2017 [17]; Ali, Ain, and HuanHe, 2020 [18]). There are several approaches of nanofibers production such as phase separation, consisting of joint gelation of the polymer and solvent (Ma and Zhang, 1999 [19]). Another technique is molecular self-assembly, which combines individual molecules based on the action of intermolecular forces (Xu, Samways, and Dong, 2017 [20]). During template synthesis polymer is pressed into a special corundum template, which allows obtainment of nanofibers of a defined shape (Wang et al., 2010 [21]). The technology, which has been intensively developed in recent years, known as electrospinning, deserves special attention. This method uses an electrostatic field to obtain fibers with a diameter of a few nanometers. Compared to other methods, electrospinning is cheaper and has a wide range of materials from which nanostructures can be spun. Nanofibers prepared by the electrospinning method have been widely used in many different applications, such as tissue engineering, cosmetics industry, filtration processes, and wastewater treatment (Huang et al., 2003 [22]; Bhardwaj and Kundu, 2010 [23]), as well as microbes adsorption process for different usage. For example, Bao et al. used polyethersulfone nanofibers for simultaneous removal of dyes and bacteria (Bao et al., 2021 [24]), and Jayani et al. used bacterial cellulose nanofibers prepared by the electrospinning method as a carrier material for *Lactobacillus acidophilus* 016 (Jayani et al., 2020 [25]). However, so far no report could be found for the usage of nanofibers—alginate hydrogel layered structure, as support for bacteria immobilization.

In this study, the layered structure of alginate hydrogel beads with immobilized bacteria cells and polystyrene nanofibers was prepared. The alginate hydrogel were prepared with gravitational dropping 2% sodium alginate solution from syringe into 1% calcium chloride solution and then sandwiched with two PS mats via electrospinning process. This layer-by-layer immobilization process was used for improving the stability, chemical, and thermal resistance, as well as reduce the leaching of entrapped bacteria. Moreover, the viability of probiotic bacteria was determined, as well as UV resistance.

## 2. Materials and Methods 

### 2.1. Bacteria Cultivation and Immobilization on Electrospun Material

In the research the bacteria strain *Lactobacillus plantarum* (PCM 2675, Polish Collection of Microorganisms, Wrocław, Poland), which is typically representative of probiotic bacteria. It has been extensively characterized in the literature and has been used in biotechnology. Hence, finding possibilities for its effective immobilization and protection will be very important for producers and also, indirectly, for consumers. The first step in the preparation of the bacterial culture was the establishment of the inoculum of the strain under study. For this purpose, the biomass from Petri dish was transferred from the solid medium to 45 mL of MRS broth (BTL Poland, Łódź, Poland) using a microbiological loop and incubated for 24 h at 30 °C with shaking (120 rpm). Then, all the bacterial inoculum and 1.5 mL of Tween 80 were added to the 1.5 l sterile MRS broth previously prepared in the bioreactor. The bioreactor (Biostat B plus, Sartorius, Germany) operating parameters, maintained throughout the culture, were set as follows: 25 °C, stirring using a Rushton turbine type stirrer 150 rpm, culture running time 6 days. Afterward, the bacterial culture was transferred to bottles and centrifuged (20 min, 4 °C, 4500 rpm). The biomass was resuspended in 100 mL of PBS buffer to the optical density OD_600_ around 2.0, and used for the immobilization procedure. Freeze-dried bacteria and sodium alginate (3%) immobilized bacteria were used as a comparison in the experiments. For freeze-drying, 2 mL of bacterial suspension was placed in a lyophilizer (Alpha 1–2 LD plus, Christ, Germany) for 96 h, −30 °C, 0.36 mbar. For bacteria immobilization in sodium alginate (SA), 2 mL of bacterial suspension was mixed with equal volume of 3% SA. The beads were formed by dropping the mixture to 200 mM CaCl_2_ solution.

### 2.2. Production of Electrospun Fibers with Encapsulated Bacteria

**Figure 1:**
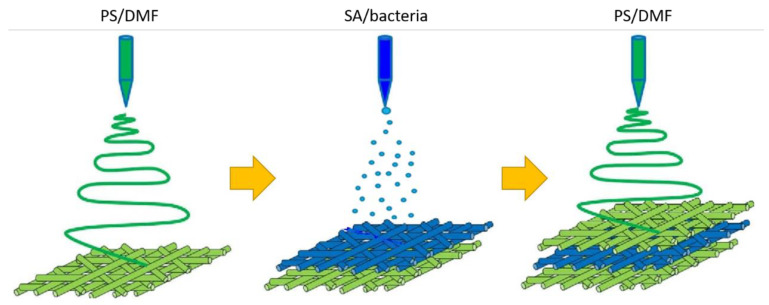
Biocomposite preparation—a workflow scheme.

Polystyrene (PS, average molecular weight 192,000 g mol^−1^, Sigma-Aldrich, Warsaw, Poland) was dissolved in *N*,*N*-dimethylformamide (DMF, Sigma-Aldrich, Warsaw, Poland) to obtain a 32% (*w*/*v*) solution and next was mixed for 24 h. After that 500 µL of Pluronic^®®^ P-123 (Sigma-Aldrich, Warsaw, Poland) was added to increase the hydrophilicity of PS electrospun fibers and mixed for another 2 h. At the same time, sodium alginate was dissolved in water (24 h) to obtain a 3% of SA solution. Subsequently, 2 mL of bacteria solution (OD600 ≈ 2.0) was added to the 2 mL of SA solution thoroughly. The prepared PS/DMF and SA/bacteria solutions were placed separately into syringes with needle sizes of 20 G and 22 G, respectively. The production of electrospun fibers from PS layered with bacteria encapsulated into SA was carried out using electrospinning and electrospraying techniques, respectively. Alternately, electrospun polystyrene (PS) fibers and SA/bacteria electrospray were layered to obtain “sandwich-structure” material consists of three layers of PS fibers and two layers of encapsulated bacteria into SA, in three repetitions (Figure 1). Both outer layers of the material were made from polystyrene.

The electrospinning process of PS was carried out under the following conditions: applied voltage 14 kV, feed rate 1 mL h^−1^, the distance between nozzle and collector 15 cm, time of process 2 min. The electrospraying conditions were: applied voltage 25 kV, feed rate 0.5 mL h^−1^, the distance between nozzle and collector 15 cm, time of process 10 min. The fibers with bacteria encapsulated were collected on the aluminum foil, which covered the steel collector and dried for 24 h in a vacuum drier at 23 °C and 50 mbar (Memmert, Schwabach, Germany).

### 2.3. Material Properties Analysis

Confocal laser scanning microscopy (CLSM) photographs of the PS electrospun material with or without bacteria were obtained with the LSM710 microscope (Zeiss, Jena, Germany) equipped with the argon laser (laser operated at 453 nm for reflected light, and 488 nm for fluorescence mode with the fluorescence observed in the range 510–797 nm).

A scanning electron microscope (SEM) (Hitachi S-3400N, Tokyo, Japan) was applied to observe the surface morphology of the PS/SA/bacteria composite. The average diameter of the PS electrospun fibers was calculated using ImageJ National Institute of Health software (1.53, Bethesda, MD, USA) from 100 points randomly selected from the CLSM images.FT-IR analyses were performed on a Vertex V70 FT-IR spectrometer (Bruker Optik GmbH, Leipzig, Germany) further equipped with a Platinum-ATR-unit (Bruker Optik GmbH, Leipzig, Germany), The material was placed onto the ATR crystal and scanned. To analyze recorded IR spectra the OPUS (7.2, Bruker Optik GmbH, Leipzig, Germany) software was used.

The contact angle was calculated based on measurements made of contact angles of water, LB broth, and milk drops on the surfaces of the materials with the accuracy ±0.01 mN/m using a Drop Shape Analysis System DSA100E (KRÜSS GmbH, Hamburg, Germany).

### 2.4. Evaluation of Bacterial Metabolic Activity

Immobilized probiotics were subjected to high temperature and UV light tests (UV-30A model UV lamp, Esco, Singapore, Singapore). For this purpose, from each material three 3 cm × 3 cm squares were cut and placed in the incubator at 50 °C for 24 h or under UV light (λ = 253 nm; distance to the light 45 cm; exposition time 30 min). Subsequently, materials were placed in the cell culture plate and flooded with 5 mL of sterile MRS broth. Samples were incubated for 24 h, 30 °C, 120 rpm and subjected to microbial activity analysis: MTT test and alamarBlue test. The control samples were material squares of the same dimensions without temperature or UV light treatment. The results were compared with the lyophilized bacteria and alginate (3%) encapsulated bacteria.

In the 96-well microplate, 200 μL of each tested bacterial suspension and 20 μL of MTT solution (5 mg/mL, Sigma-Aldrich, Warsaw, Poland) were mixed to measure metabolic activity. The plate was incubated for 24 h at 30 °C until formazan crystals formed. The biomass was then centrifuged and 200 µL of isopropanol was added to each well. The plates were left on a shaker with gentle mixing for about 15 min until the formazan crystals were completely dissolved, then briefly centrifuged, and the clear supernatant was transferred to a clean measuring plate and the absorbance was measured at 600 nm.

Analysis of cell metabolic activity using the alamarBlue reagent (Biorad, Warsaw, Poland) involved applying 200 μL of each bacterial culture to a 96-well plate at 3 dilutions with phosphate buffer (1:1; 1:2, 1:4), then 20 μL of alamarBlue reagent was added to each well and incubated at 25 °C with shaking, taking spectrophotometric measurements every hour at 570 nm and 600 nm for 8 h (Multiskan Sky, Thermo Fisher Scientific, Warsaw, Poland). The results were calculated as a percentage difference in the reduction of the reagent in comparison to control samples, according to Equation (1).

(1) $$M=\frac{\left(O2*A1\right)-\left(O1*A2\right)}{\left(O2*P1\right)-\left(O1*P2\right)}*100$$

where: 

*O*1 = molar extinction coefficient of oxidized alamarBlue at 570 nm (80,586 M^−1^ cm^−1^)

*O*2 = molar extinction coefficient of oxidized alamarBlue at 600 nm (117,216 M^−1^ cm^−1^)

*A*1 = test wells absorbance at 570 nm 

*A*2 = test wells absorbance at 600 nm

*P*1 = positive growth control well absorbance at 570 nm 

*P*2 = positive growth control well absorbance at 600 nm

Two hundred microliters of distilled water supplemented with alamarBlue or MTT reagent were used as blanks. For each system tested, three replicates of the experiment were performed.

## 3. Results and Discussion

### 3.1. Characteristic of the Material

#### Microscopy

**Figure 2:**
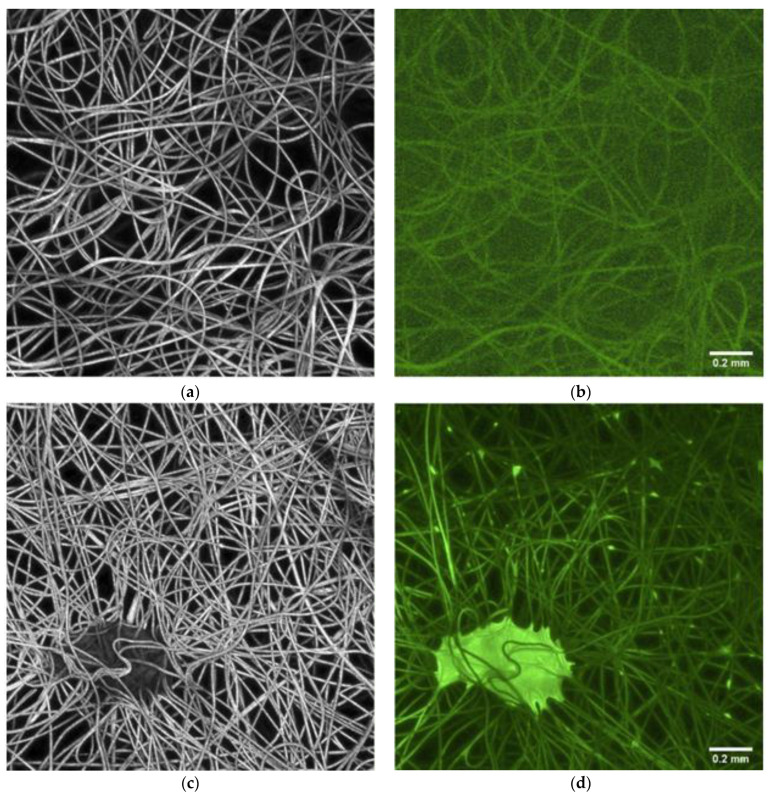
**Cross-section** CLSM pictures in normal (**a**,**c**) and fluorescence (**b**,**d**) mode of pure PS material (**a**,**b**) and PS material with alginate immobilized bacteria (**c**,**d**).

Cross-section morphology of electrospun mats and PS nanofibers with alginate immobilized bacteria (PS/Alg) were characterized based on the CLSM photographs (LSM710, Zeiss, Jena, Germany) in normal and fluorescence mode (Figure 2). Presented results confirm successful immobilization of the probiotic bacteria on the nanofibers. Untreated PS nanofibers display a smooth, uniform surface, with no fluorescence. The fibers morphology became beaded after the immobilization process, comparing it to the pure mat (Figure 2a,c). The beads of oblong shape are distributed along the fibers, with some bigger cluster spots. Moreover, a strong fluorescence of these structures confirms that they are alive probiotic cells. A similar phenomenon has been reported by a previous studies (Mamvura et al., 2011 [26]; Yu et al., 2020 [27]).

**Figure 3:**
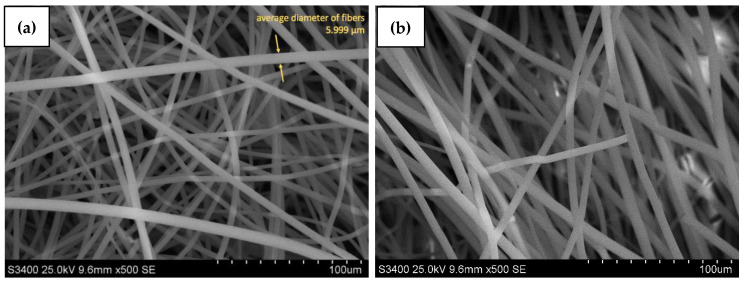
Top-view SEM pictures of PS nanofibers (**a**) and PS nanofibers with alginate immobilized bacteria (**b**) with a magnitude of 500.

Furthermore, the electrospun PS nanofibers and PS/Alg material were characterized based on the SEM images (S-3400N, Hitachi, Tokyo, Japan) after gold coating (Figure 3). Moreover, the SEM photographs were used for the calculation of the average diameters of electrospun fibers. Presented photos of the top view confirmed that the electrospinning process was successfully used for nanofibers production. Fibers did not possess any beads or entanglements which proves that and process conditions were chosen correctly. Figure 3b shows that PS/Alg material possesses intercalated alginate hydrogel particles (visible in the background, behind PS fibers). Nanofibers’ diameter is constant through the whole membrane and equals ~6 nm for both PS and PS/Alg materials. The same conclusions were drawn by Deng et al., who used electrospun polystyrene nanofibers as a mat for filtering atmospheric air from the heavy metal trace. They used the PS/DMF/THF solution for the production process, with the following electrospinning settings: voltage 20 kV, dosing speed (feeding rate) 1 mL h^−1^, and needle-collector distance 20 cm. The diameter of obtained nanofibers was 407.6 ± 118.7 nm (Deng et al., 2020 [28]).

**Figure 4:**
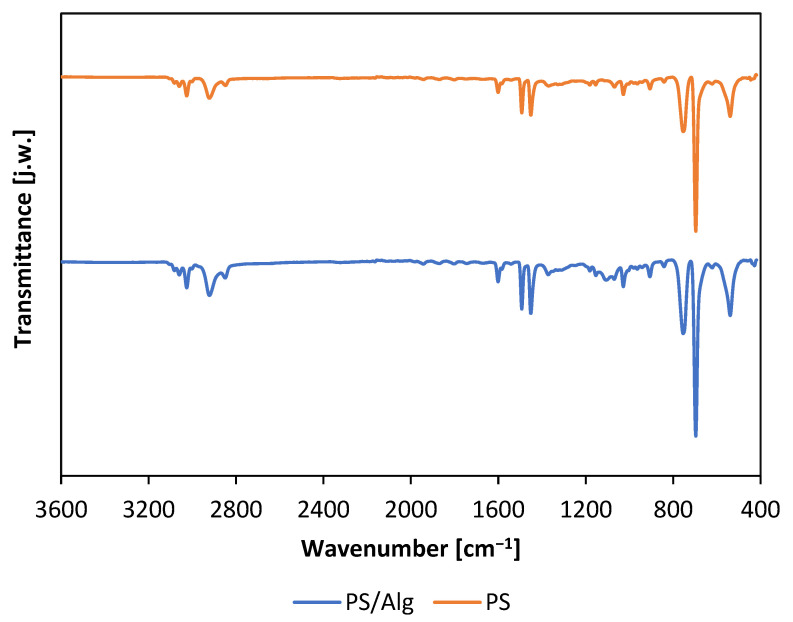
Infrared spectra of polystyrene nanofibers (PS) and polystyrene nanofibers with alginate hydrogel immobilized bacteria (PS/Alg).

To further investigate the tested materials, infrared spectra of the materials were undertaken (Figure 4). The obtained spectra clearly show signals at 1600 and 1497 cm^−1^ as well as at 758 and 699 cm^−1^ corresponding respectively to the signals from chemical bonds: C=C (stretching vibrations of benzene-rings) and the out-of-plane bending of C–H of mono-substituted benzene-ring). Moreover, the absence of signals from O–H bond vibrations is notable. Signals in the region of 3000 cm^−1^ can be attributed to C–H bond dranes in both the alkyl chain and the aromatic ring. The significant similarity of both spectra should be emphasized, which represent mainly the same nanofiber material—polystyrene, which comprises the outer layer of the PS/Alg nanomaterial. Nevertheless, the infrared spectra proved the stability of the PS nanofibers during the preparation of composite material and indicated that the surface properties of PS/Alg material are dominated by PS nanofibers properties.

**Figure 5:**
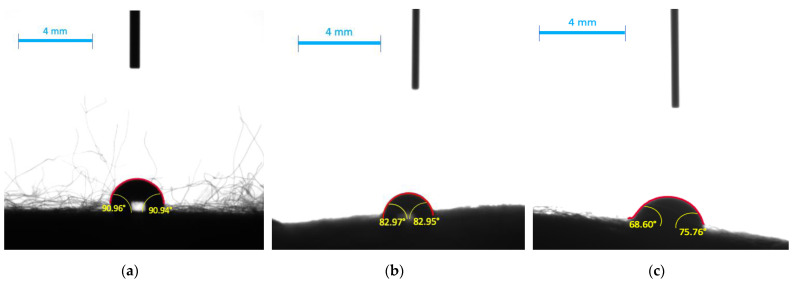
Contact angles of electrospun nanofibers from polystyrene-alginate-*L. plantarum* 2675 obtained with: (**a**) water; (**b**) MRS broth; (**c**) milk (2% fat).

Successively, the contact angles (CA) measured for electrospun fibers with the immobilized bacteria were analyzed and they are presented in Figure 5. Additionally, calculated values are collected in Table 1. Hydrophobicity of obtained material was characterized based on a measurement of contact angle (DSA100E Drop Shape Analyzer, KRÜSS GmbH, Hamburg, Germany). In general, materials with liquid drop CA on their surface greater than 90° are hydrophobic, and with CA lower than 90° are hydrophilic. PS/Alg nanofibers showed slightly hydrophobic (CA > 90°) properties when a drop of water was applied. This is directly connected to polystyrene hydrophobic properties and was not affected by the presence of sodium alginate hydrogels. Moreover, the presence of alginate decreases the CA in the material, as we can observe higher values of CA for PS than for PS/Alg.

These result correspond to results presented by Huan et al. who obtained water CA 106 ± 1.5° and 90 ± 1.2° when electrospinning PS nanofibers from DMF and THF respectively (Huan et al., 2015 [29]). The lower contact angle was obtained for broth and milk and equals 83.0 ± 3.7° and 72.2 ± 2.89°, respectively, which means that both liquids possess the higher wetting ability. This could be caused by the presence of additives like proteins, sugars, and lipids, of which some are surface-active agents responsible for increasing wetting ability (Lorentz, Rogers and Jones, 2007 [30]; Campos Bernardes et al., 2012 [31]). A similar conclusion was drawn by Handojo et al. who reported that CA for water was higher than for whole milk on a glass surface and was equal to 49.5 ± 3.1° and 21.0 ± 4.7°, respectively (Handojo et al., 2009 [32]).

### 3.2. Bacterial Metabolic Activity

**Figure 6:**
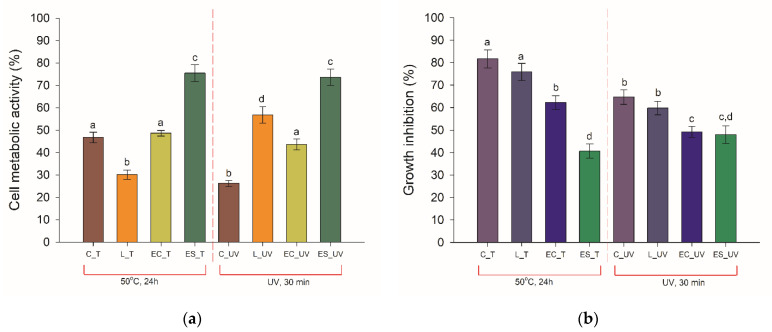
Results of the measurements of: (**a**) cells’ metabolic activity, (**b**) growth inhibition of the cells, performed for control (C), lyophilized (L), alginate hydrogel-encapsulated (EC), and electrospun fibers immobilized (ES) cells of *L. plantarum* 2675 exposed to increased temperature (50 °C, 24 h) or UV radiation (λ = 253 nm, 30 min). Error bars represent the standard error of the mean of the three replicates in three independent experiments. Different lowercase letters suggest the significant differences among mean values for each treatment group (*t*-test; *p* < 0.05).

Probiotic bacteria cells’ survivability is challenged by heat processing methods, compromising their functionality. To enhance the thermal stability of cells, various immobilization techniques are applied. For this purpose, the analysis of the metabolic activity of bacteria immobilized into the produced polystyrene electrospun nanofibers/alginate hydrogel was performed. The metabolic activity of the cells was measured based on the MTT and alamarBlue assays, comparing the obtained results with traditionally preserved probiotics (lyophilized and encapsulated in sodium alginate). The results collected in Figure 6a present a decrease of cells’ metabolic activity upon 24 h exposition to elevated temperature and UV radiation, regardless of preservation technique. However, the electrospun-fibers immobilized cells possessing the highest metabolic activity of the analyzed samples. The type of the factor influencing cells was also meaningless, which proves good protective properties of the produced fibers.

Moreover, these results are also in consistency with the alamarBlue test results which measures cellular respiration during proliferation. In this case, the electrospun fibers immobilized bacteria were characterized with low growth inhibition, equal to 40% and 48% by the applied temperature and UV light, respectively (Figure 6b). At the same time, the growth of free cells was inhibited by 81% (exposure to higher temperature) and 64% (exposure to UV radiation), and a significant decline in growth was observed for other analyzed samples. The protective effect of the matrix surrounding the cells on bacterial proliferation properties was especially visible when the UV light was applied. The free and lyophilized cells showed over a 60% decline in growth, while for immobilized cells it was around 10% lower.

The protection efficiency of *L. plantarum* 2675 cells by the produced membrane was investigated proving increased cell metabolic activity and growth under heat and UV radiation treatment in bath, MTT, and alamarBlue tests. Better thermal stability of the *Lactobacillus* cells encapsulated in the core–shell fiber mat (PVA) was noted by Feng et al. (2020) [33], with the viability loss of 0.32 log(CFU mL^−1^) after 30 min in 70 °C. As suggested earlier by Feng et al. (2018) [34], the high capacity of the produced PS nanofibers might be related to the shielding effect of the nanomaterial, thus physically inhibiting the hot diffusion into the material. The combination of polymer and hydrogel was successfully applied by Gensheimer et al. (2011) [35]. They immobilized the *E. coli* and *M. luteus* cells in PVA-hydrogel and obtained microparticles that were linked with PLA, PVB, or PCL nanofibers. However, the authors tested only protection against organic solvents. Hence it is hard to compare their results with ours. Moreover, it should be underlined, that the review prepared by Stojanov and Barlec (2020) [36] includes the most frequently used polymers used in nanofibers production for cell immobilization and does not mention broader data about polystyrene. Our study can be considered as a valuable contribution to the present state of knowledge as a proof of usability of the PS nanofibers with alginate hydrogel to immobilize bacteria. The method can be applied mainly for Gram-positive bacteria with cell structure similar to *Lactobacillus* cells. For other groups of bacteria, Gram-negative, the effectiveness of the hybrid material should be similar. However, because of the differences between cell walls of Gram-positive and Gram-negative bacteria, for the second group additional experiments must be conducted.

## 4. Conclusions

Increasing demand for safe, efficient, and environmentally-safe solutions for pharmaceutical and food industries force researchers to look for new methods of bacteria storage. We propose that composite polystyrene materials/alginate immobilized bacteria meets mentioned expectations, since the research has proved the stability of the hybrid material. Significant reduction of the toxic effect of UV radiation and the higher temperature (50 °C, 24 h) was observed when the bacteria were immobilized in composite in comparison with free cells and cells in alginate only. Good wettability properties make the hybrid material a promising tool for industrial applications.

## Figures and Tables

**Table 1:** Contact angles values of tested materials with selected dietary liquids.

Material	Medium	Left Angle [°]	Right Angle [°]	Average Angle [°]
PS	Water	97.10 ± 3.6	97.10 ± 3.6	97.10 ± 3.6
	Broth	97.23 ± 1.0	97.23 ± 1.0	97.23 ± 1.0
	Milk (2% fat)	77.24 ± 2.2	77.24 ± 2.2	77.24 ± 2.2
PS/Alg	Water	91.0 ± 2.9	90.9 ± 3.3	91.0 ± 3.1
	Broth	83.0 ± 3.3	83.0 ± 4.1	83.0 ± 3.7
	Milk (2% fat)	68.7 ± 0.3	75.8 ± 5.5	72.2 ± 2.9

## Data Availability

No new data were created or analyzed in this study. Data sharing is not applicable to this article.
